# Surgical Determinants of Outcomes in Partial Nephrectomy: a Contemporary Review

**DOI:** 10.1007/s11934-026-01329-0

**Published:** 2026-04-06

**Authors:** Rodrigo Guevara, L. G. Medina, J. P. Dugarte, F. Eskenazi, R. Cervantes, V. Hevia, R. Sotelo

**Affiliations:** 1 Department of Urology, Keck Medicine of USC, Los Angeles, CA USA; 2 Department of Urology, Hollings Cancer Center, Medical University of South Carolina, Charleston, SC USA; 3 Department of Urology, Centro Medico ABC, Mexico City, Mexico; 4 Kidney and Retroperitoneum Division, ROC Clinic, HM Hospitales, Madrid, Spain

**Keywords:** Partial nephrectomy, Nephron-sparing surgery, Renal cell carcinoma, Tumor complexity, Ischemia strategies, Surgical outcomes

## Abstract

**Purpose of Review:**

Partial nephrectomy has become the preferred treatment for localized renal masses, particularly in the minimally invasive surgery (MIS) era. However, outcomes depend not only on the surgical approach but also on how each step of the procedure is performed. This review examines the key technical steps and clinical determinants that influence oncologic control, renal functional preservation, and perioperative morbidity in contemporary minimally invasive partial nephrectomy.

**Recent findings:**

Recent evidence highlights the importance of assessing tumor complexity, tailoring ischemia strategies, and selecting resection techniques that maximize parenchymal preservation. Advances in intraoperative imaging, three-dimensional planning, and fluorescence guidance have improved surgical precision and supported more informed intraoperative decision-making. Comparative studies also suggest that techniques such as tumor enucleation and selective clamping can help preserve renal function without compromising oncologic outcomes in appropriately selected patients.

**Summary:**

Current evidence suggests that outcomes after PN depend largely on tumor characteristics, patient factors, and the way the operation is performed, rather than on the surgical platform itself. Achieving optimal results requires careful preoperative planning, precise surgical technique, and adequate experience. Ongoing advances in imaging integration and intraoperative guidance technologies may further support surgical decision-making and help improve renal functional preservation in nephron-sparing surgery (NSS).

## Introduction

Partial nephrectomy (PN) was introduced in the late nineteenth century [1]. Despite its relatively slow initial adoption, PN demonstrated oncologic equivalence to radical nephrectomy (RN) and progressively evolved from a procedure reserved for imperative scenarios to the standard of care for small renal masses [1–4]. Furthermore, advances in surgical instrumentation, imaging, ischemia control, renal reconstruction, hemostasis, and a better understanding of the small renal masses biology have reinforced the principles of NSS [1–3, 5].

During the minimally invasive era, not only have PN surgeries increased threefold, but RN rates have decreased twofold [6]. Furthermore, robot-assisted partial nephrectomy (RAPN) has become the most predominant approach in US. Practice [7] with similar adoption trends reported in Spain, Japan and UK, reflecting the global expansion of robotic platforms for NSS [8–10]. Large observational studies have demonstrated the advantages of the Minimally invasive surgery (MIS) approach over the open approach, including reduced ischemia time, reduced blood loss, and a faster return to baseline functional status [11–15]. Despite the current predominance of minimally invasive approaches, open PN remains a valid option in selected scenarios, particularly for tumors of very high complexity, central masses with extensive hilar involvement, complex reoperative fields, or when major reconstruction is anticipated [16, 17].

With the widespread adoption of MIS as the standard of care for renal tumors, focus has shifted from the selection of surgical approaches to refining how the procedure is performed. Evidence suggests that surgical outcomes depend less by the approach than by the technical aspects of each procedure step [18]. Aspects such as plane of resection, ischemia control, parenchymal preservation, intraoperative imaging, and renal reconstruction play a significant role in optimizing oncologic, functional, and morbidity outcomes [6, 11, 12]. Although these technical aspects have long been discussed, they are often discussed in isolation.

In this review, we synthesize the latest evidence across the key surgical determinants of outcomes in PN. Our goal is to provide a unified, evidence-based framework to optimize oncologic, functional, and perioperative outcomes in modern minimally invasive PN.

## Historical Evolution of the Partial Nephrectomy

In 1861, the first total nephrectomy was inadvertently performed during a liver cyst excision leading to two discoveries: a kidney could be safely excised if needed, and the fact that a person could survive with only one kidney [1]. In 1884, Wells described a PN technique for kidney fibrolipomas, later applied by Czerny to renal malignancy [19]. However, due to increased morbidity, its use was reserved for benign conditions [1, 2].

By 1937, a retrospective series reported favorable perioperative outcomes in PN, but its wider adoption remained limited until Vermooten proposed in 1950 that small cortical tumors could be safely managed with PN [1, 20]. The resurgence of PN occurred in the 90s, when a large series demonstrated low local recurrence rates with survival of 95–100% [1, 2].

MIS as an alternative for PN was first introduced by McDougall using pig models [21]. Subsequent large series by Haber and Gill confirmed the feasibility of laparoscopic PN, although its adoption remained limited because of a steep learning curve [22].

The EORTC 30,904 trial demonstrated that NSS significantly reduces the incidence of moderate chronic kidney disease (CKD)(eGFR < 60) compared with RN in patients with small renal masses. Specifically, after a median follow-up of 6.7 years for renal function, the incidence of eGFR < 60 was 64.7% after NSS versus 85.7% after RN, confirming a functional benefit in terms of nephron preservation with similar long-term oncologic outcomes [23, 24].

The RAPN was introduced in 2003 [25]. Soon thereafter, MIS PN approach was rapidly adopted, and its indications expanded to include larger and more complex tumors [26–28].

Comparative studies and meta-analysis have shown equivalent oncologic outcomes among approaches, with advantages for MIS in blood loss, transfusion rates, hospitalization, complications, and functional outcomes [27, 28]. More recently, the evolution of robotic platforms has extended to single-port (SP) systems aimed at further reducing surgical invasiveness while maintaining the advantages of robotic surgery [29].

## Tumor and Host-Related Factors

### Tumor Complexity

Preoperative planning for a PN, traditionally begins with an evaluation of anatomic complexity based on established nephrometry systems after cross-sectional imaging. The R.E.N.A.L [30]. and PADUA scores [16] continue to serve as widely utilized instruments to stratify case difficulty, perioperative risk estimation and guide ischemia planning in both laparoscopic and robotic platforms, especially for intermediate and complex masses where nephrometry positively correlates with blood loss, operative time and conversion risk [16, 31, 32]. Therefore, hilar tumors, due to their proximity to the renal sinus, are associated with higher rates of conversion to RN. Likewise, more posterior tumors, although not associated with increased conversion rates, may require more extensive mobilization of Gerota’s fascia, usually increasing operative time, depending on the selected surgical approach [33]. Both systems also facilitate identifying when technical difficulty and complication rates increase [16, 31, 32].

Recent studies suggest that higher nephrometry scores are independently associated with higher perioperative complications rates and postoperative AKI after RAPN [31, 34, 35].

The use of three-dimensional reconstructions and volumetric planning are increasingly used, especially for posterior, hilar, and deeply endophytic tumors; 3D planning can improve understanding of arterial anatomy, collecting system relationships, and perfusion territories, facilitating selective or supraselective strategies and allowing estimation of preserved parenchymal volume [36–39]. Physical 3D printing has also been associated with reductions in ischemia time and bleeding in selected cohorts [40], and augmented-reality (AR) overlays may improve intraoperative orientation in complex masses [37]. In addition to anatomic geometry, evidence suggests that parenchymal volume preservation is a major determinant of long term renal functional outcomes after PN, which supports preoperative volumetric assessment and individualized planning [41, 42].

Recent studies have been explored immunotherapy and targeted agents in perioperative settings for renal cell carcinoma (RCC), especially in locally advanced or surgically complex tumors. The PADRES study which assessed 8 weeks of neoadjuvant axitinib in complex localized clear cells renal cell carcinoma (ccRCC) showed a median tumor shrinkage of approximately 17%, with a 33% partial response rate by RECIST and partial nephrectomy feasible in 74% of cases. In contrast, KEYMAKER-U03 (substudy 3 A), evaluated immune checkpoint inhibition–based combinations including pembrolizumab and the HIF-2α inhibitor belzutifan in treatment-naïve advanced ccRCC, demonstrating high RECIST response rates in the advanced disease. Nevertheless, include heterogeneous response, systemic toxicity, surgical timing considerations, and the lack of randomized data supporting long term oncologic benefit over upfront surgery [24]. 

### Tumor Size and Location as Determinants of Strategy

Tumor complexity, driven by size, endophytic growth, sinus/hilar proximity, and collecting system involvement, should guide intraoperative decision-making [16, 31, 32]. Hilar tumors represent the clearest example: proximity to major vessels and renal pelvis increases operative risk and often justifies full hilar control to ensure a bloodless field and precise reconstruction [32, 43]. Importantly, even when reduced-ischemia or off-clamp strategies are contemplated, early and systematic dissection of the renal hilum is recommended to allow immediate vascular control if required, reinforcing that hilum preparation is a safety prerequisite rather than a commitment to clamping [44]. In this setting, attempts at no-clamp resection have been associated with higher transfusion rates and increased conversion to RN without a consistent functional advantage [1, 32, 45].

Nonetheless, highly selected complex tumors may be managed with reduced ischemia strategies in expert hands [39, 46]. Importantly, CLOCK data emphasize that intraoperative transition from off- to on-clamp is not rare and should be anticipated as a safety mechanism rather than viewed as failure; predictors of crossover include anatomy and tumor-related factors that become evident intraoperatively [43]. This aligns with broader conversion literature in RAPN showing that complex anatomy, bleeding, and adverse hilar events remain the principal triggers for escalation of strategy, including conversion to RN or open surgery to preserve patient safety [33].

### Selection of the Surgical Approach

In selecting the surgical access route, both transperitoneal and retroperitoneal approaches provide comparable oncologic and functional outcomes. The transperitoneal route may remain preferable for anterior masses (especially when using multiport platforms), hilar tumors requiring broader working space, or when surgeon experience favors this route [32, 47, 48].

In systematic reviews and propensity-matched analyses, retroperitoneal multiport RAPN has been associated with shorter operative time, reduced blood loss, shorter length of stay, and lower overall complication rates, advantages that are most relevant for posterior/posterolateral tumors where direct access to the hilum simplifies dissection [47, 48].

The development of SP robotic technology has introduced new technical variants aimed at reducing parietal trauma and improving recovery without compromising oncologic control. In contemporary series and comparative analyses, SP PN demonstrates similar outcomes to multiport RAPN regarding positive margins, major complications, and short-term functional preservation, with no differences in ischemia time (29.6 SP vs. 28.8 MP, *p* = 0.008) and same transfusion rates (OR 2.99–95% CI 1.31–6.80). These findings may vary according to patient selection and surgeon experience [38, 49–51]. More recently, the introduction of a supine anterior retroperitoneal access (LAA (Low anterior access)/ SARA (supine anterior retroperitoneal approach)) has been proposed as a technical refinement specifically tailored to SP platforms, aiming to simplify retroperitoneal access, improve ergonomic orientation, and reduce the technical barriers associated with the traditional lateral flank approach. Importantly, the ability of SP platforms to expand indications toward higher complexity remains under active evaluation; current evidence supports feasibility but does not yet establish a consistent functional advantage over conventional multiport RAPN [38, 49, 51]. Moreover, the available SP-RAPN evidence is less mature than that of multiport platforms. Most published SP-RAPN series also predominantly include small (≤ 3 cm) and low-complexity renal tumors. While early series with SP-RAPN using SARA demonstrate safe and reproducible perioperative outcomes with adequate hilar exposure in selected renal tumors, these data derive from limited, noncomparative experiences and should be interpreted as proof of concept rather than evidence of superiority [51, 52].

### Host-Related Factors

Host factors including obesity, advanced age, and baseline CKD, modulate perioperative risk and functional recovery and should be integrated into preoperative counseling and planning. Specifically, obesity has been associated with longer operative times (mean increase 13 min), greater estimated blood loss (45 mL), and a significantly higher risk of postoperative complications (odds ratio 1.5), reflecting increased technical difficulty related to exposure, perinephric fat, and reconstruction demands. Baseline CKD confers a significantly increases perioperative risk, with nearly doble the rate of postoperative complications and reduced functional reserve, limiting the kidney’s ability to tolerate ischemic or parenchymal injury. Advanced age further adds complexity due to lower physiologic reserve and a higher burden of comorbidities, which may impair functional recovery even when oncologic and technical outcomes are satisfactory [53, 54].

In patients with impaired baseline renal function or more advanced CKD, PN can still be justified in selected cases, but the margin for error narrows and the importance of minimizing avoidable ischemic injury and preserving vascularized nephron mass becomes paramount [42, 54]. In patients with stage IV CKD, reported major adverse events rates approaching between 20 and 25%, including significant perioperative complications and progression to end-stage renal disease. This emphasizes the need for careful selection when offering PN in this high-risk population [53, 54].

## Surgical-Related Factors

### Hilar Control and Resection Technique

Intraoperative strategy during PN directly influences both oncologic safety and functional preservation. Decisions regarding hilar control and resection technique should be driven primarily by tumor complexity and anatomy rather than by an attempt to minimize ischemia [32, 34, 43]. An important concept to frame these choices is that surgically induced renal dysfunction is distinct in terms of biologically and prognostically from medical CKD, which reflects chronic systemic disease and is more commonly associated with adverse long-term outcomes in the surgical setting [43] Consequently, the priority is not simply “zero ischemia,” but the safest route to maximize vascularized renal parenchyma while maintaining oncologic precision and hemostatic control [41, 43].

Conventional hilar clamping remains the most reproducible and broadly applicable strategy, particularly in complex tumors, providing optimal visualization and hemostatic control during excision and reconstruction [32, 34, 43]. Contemporary evidence supports the clinical relevance of warm ischemia time (WIT), with increasing ischemia associated with worse short-term renal function, and with commonly cited cautionary thresholds beyond approximately 25–30 min [34, 43]. However, an exclusive focus on WIT can be misleading as the amount of renal parenchyma preserved is typically a stronger predictor of renal function than small differences in ischemia time [41, 43].

In a systemic review and meta-analysis including 1,113 patients, focused on laparoscopic series, renal artery only vs. renal artery plus renal vein (AV) clamping showed no significant differences in WIT, surgical time, transfusions rates and other functional outcomes. Likewise, AV clamping was associated with a greater percentage decline in eGFR at last follow-up (WMD 2.42, *p* < 0.00001). Recent studies have shown that RAPN for moderate to high complexity renal masses achieve comparable WIT and no significant differences in blood loss, AKI, or renal function at 1, 3 and 12 months, supporting AV clamping when venous bleeding control is a concern. Clamp selection should be individualized to tumor complexity and surgeon preference [55, 56].

Early unclamping has been proposed to reduce WIT. In the CLOCK trial, no statistically significant differences were found between clamping strategies in terms of renal function (*p* > 0.05) or in the incidence of AKI (*p* > 0.05), whereas in the EMERALD study, a single-blind trial comparing ICG near-infrared fluorescence-guided supraselective clamping with conventional hilar clamping plus early unclamping did not demonstrate superior long-term renal function at 6 months (split eGFR change − 21.4% vs. −23.4%; *p* = 0.66) [39]. Therefore, early unclamping is an attractive concept and is technically reasonable during PN reducing WIT; variability of renal vasculature and tumor perfusion arising from different branches may limit its use, as there is no proven durable functional benefit [39, 43, 57].

Zero-ischemia concepts include off-clamp resection as well as anatomically guided devascularization supported by indocyanine green (ICG) fluorescence [39, 56]. Although technically appealing, purely off-clamp approaches shift the primary risk from ischemia to bleeding control and intraoperative instability. Large and multicenter datasets have demonstrated no consistent functional superiority of off-clamp RAPN, while reporting higher transfusion rates and increased conversion to RN [1, 46, 57]. Randomized data in laparoscopic PN (CLOCK II) similarly demonstrate that off-clamp and on-clamp approaches demonstrate comparable perioperative outcomes in appropriately selected small renal masses, reinforcing that off-clamp is not definitively superior and remains context-dependent [31]. From the authors perspective, zero-ischemia should be considered an advanced option best reserved for favorable anatomy and high-experience settings rather than a default objective [1, 39, 43, 45].

WIT can induce ischemic renal injury when sustained for > 30 min, when prolonged ischemia is anticipated, regional hypothermia can be achieved. Laparoscopic cold ischemia was first described by Janetschek et al. in 2004 using renal artery cold perfusion, by occluding the renal artery and perfusing iced Ringer´s lactate at 4 °C trough an angiocatheter, maintaining renal temperature in the 20–25 °C range is easier than deeper hypothermia and can provide substantial protection from ischemia for at least 90 min. Open surgery data suggest that clamp times up to 58 min under cold ischemia can be comparable to 25 min of warm ischemia. Contemporary robotic techniques have used intracorporeal ice slush delivery through a 12 mm trocar using a syringe, packing the slush around the kidney containing it with laparoscopic sponges to achieve regional cooling while the hilum is clamped [58–60].

### Enucleation versus Anatomic Resection

Standard resection removes the tumor with a parenchymal margin (0.5–1.0 cm), whereas enucleation removes the tumor with no surrounding healthy tissue staying on the plane between the tumor pseudocapsule and adjacent parenchyma, associated with shorter WIT and lower eGFR decline [46, 61–65]. Although tumor enucleation is considered safe in T1 tumors, some authors suggest that in cases with suspected unfavorable histology or infiltrative tumors, a standard resection may be less safe than taking surgical margins beyond 0.5 mm. However, the evidence has not demonstrated a benefit of extending this surgical margin compared with performing a standard surgical margin [66, 67]. Comparative evidence supports that enucleation is non-inferior to standard resection in oncologic outcomes while reducing parenchymal loss [41, 43, 61]. In the systematic review and meta-analysis conducted by Xu et al., tumor enucleation was associated with shorter operative time (MD − 28.46 min), lower estimated blood loss (MD − 59.90 mL), fewer postoperative complications (OR 0.65), and preservation of renal function (MD − 3.72 mL/min/1.73 m²), with no significant differences in positive surgical margins, local recurrence, or survival compared with standard PN [66] .

From an author perspective, enucleation is oncologically sound when the pseudocapsule plane is clearly identifiable and respected, and when conversion to a wider resection is performed promptly if the plane is uncertain. This principle is particularly relevant in anatomically complex settings, as demonstrated in a propensity score matched analysis of completely endophytic tumors, where enucleation achieved a markedly smaller early decline in renal function (4.9% vs. 16%) and shorter operative time, while maintaining comparable WIT, complication rates, and margin status relative to standard resection [66, 68]. Pathologic analyses of salvage nephrectomies for ipsilateral recurrence suggest failures can relate to incomplete excision at the primary site underscoring the importance of plane control and intraoperative judgment in cases without a clear capsular interface.

### Comparative Outcomes: Functional Preservation and Complications

Intraoperative choices regarding hilar control and resection technique have direct impact on the principal outcomes, preservation of renal function and oncologic control.

Renal functional preservation depends on both ischemic injury and the volume of preserved parenchyma. While limiting WIT remains desirable, long-term renal function appears more strongly influenced by preserved, vascularized nephron mass than by ischemia time alone in many contemporary cohorts [41, 43, 69]. This distinction becomes clinically relevant when interpreting postoperative trajectories: AKI is common after PN, and both its occurrence and duration independently influence the probability of functional recovery and CKD upstaging at 1 year [35]. Therefore, perioperative renal protection should include not only ischemia management, but also meticulous hemostasis, avoidance of nephrotoxins, and strategies to minimize prolonged postoperative injury [35, 42].

In a multicenter study including 2549 RAPN, the conversion rate was 3.5%, dependent on tumor complexity and lower surgeon experience [33]. This also leads us to the learning curve, in which Wu et al. reported shorter operative time (115 vs. 150 min, *p* < 0.001) and lower WIT (19 vs. 24 min, *p* < 0.001) after 35–40 cases [70].

Bleeding remains the most frequent complication associated with off-clamp and some zero-ischemia strategies. Although mean blood loss and transfusion rates may be similar, delayed vascular complications such as pseudoaneurysm or arteriovenous fistula (AVF) can be more frequent [1, 39, 45, 71]. In a meta-analysis conducted by Cacciamani et al., RAPN was associated with lower major postoperative complications (Clavien ≥ III) compared with open surgery (OR 1.55, 95% CI 1.27–1.90; *p* = 0.0001) and laparoscopic surgery (OR 1.50, 95% CI 1.19–1.89; *p* = 0.0006). Additionally, conversion rates were significantly lower (OR 2.61, 95% CI 1.11–6.15; *p* = 0.03). In the same meta-analysis, overall mortality was lower in the robotic group compared with both open and laparoscopic partial nephrectomy; however, mortality remained low across all groups [72].

Renal artery pseudoaneurysm (RAP) is a rare but serious complication following PN. In a cohort study of 544 patients, was reported an incidence of 2.6%, with higher RENAL scores related to the requirement for embolization, being mainly driven by the N component (tumor nearness to the collecting system or renal sinus; *p* = 0.031). In comparison, the PADUA score showed no significant association with RAP. Longer time of surgery was also related with RAP development (350 vs. 284 min; *p* = 0.046) [73].

These data suggest that adequate tumor staging, selection of the surgical approach, clamping strategies, and surgeon experience may directly impact outcomes, with increasing experience, shorter ischemia times and fewer complications.

##  Intraoperative Imaging and Assessment

In the renal surgery field, Indocyanine green (ICG) fluorescence was first applied by Golijanin in 2007 in a pilot series of 10 patients undergoing nephrectomy (2 radical and 8 partial). In this cohort, all tumors appeared hypo or non-fluorescent compared to healthy parenchyma, allowing for sharp demarcation. Postoperative pathology confirmed negative margins in all cases, with a mean margin width of 4 mm, supporting the feasibility of fluorescence guidance for renal mass excision [74].

Complementing fluorescence-guided perfusion assessment, intraoperative renal ultrasonography (IORUS) has a longer history as an imaging aid in PN. IORUS was first explored more than three decades ago, when early improvements in probe miniaturization and image resolution made real-time intrarenal imaging feasible. In the first series, in 1991, Assimos et al. evaluated the use of IORUS in 6 patients undergoing PN for renal cell carcinoma and in 14 RN specimens subjected to simulated PN. With IORUS used to define tumor extent and location, negative surgical margins were achieved in all 6 clinical PN and in 13 out of the 14 simulated resections, demonstrating its potential value in localizing deep intraparenchymal lesions and aiding in obtaining negative margins [75].

These technical and practical benefits are consistent with findings from a recent retrospective study of 152 patients undergoing open or RAPN, in which IORUS was preferentially used in anatomically complex situations such as solitary kidneys, recurrent or multifocal tumors, yet was associated with low and comparable positive margin rates (3.2% vs. 1.7% without IOUS) and no increase in complications [76–78].

IOUS provides real-time imaging of tumor localization, delineation of tumor borders, and confirmation of adequate ischemia. Its clinical value during PN was demonstrated in a comparative study including patients in whom IOUS was not used during surgery. In that analysis, IOUS-guided cases were associated with significantly lower estimated blood loss (144.7 vs. 257.5 mL; *p* < 0.001), shorter warm ischemia time (20.4 vs. 25.6 min; *p* = 0.010), and a smaller percentage decline in eGFR (6.4% vs. 9.9%; *p* = 0.007), without significant differences in positive surgical margin rates (7.9% vs. 15.0%; *p* = 0.405) [76–79]. 

Beyond fluorescence-guided imaging and IORUS, emerging digital technologies are further refining intraoperative decision-making. In this context, image-guided surgery (IGS) has gained relevance in RAPN. IGS refers to the use of preoperative imaging data, integrated and displayed during the procedure, to guide surgical maneuvers in real time. Emerging applications are critical to safely and successfully performing RAPN [80].

Building on this framework, Ukimura and Gill’s 2008 report provided the first clinical application of image-guided technology in laparoscopic PN, using preoperative CT-based 3D navigation models superimposed onto the real-time operative field to guide resection while maximizing parenchymal preservation [81].

**Fig. 1:**
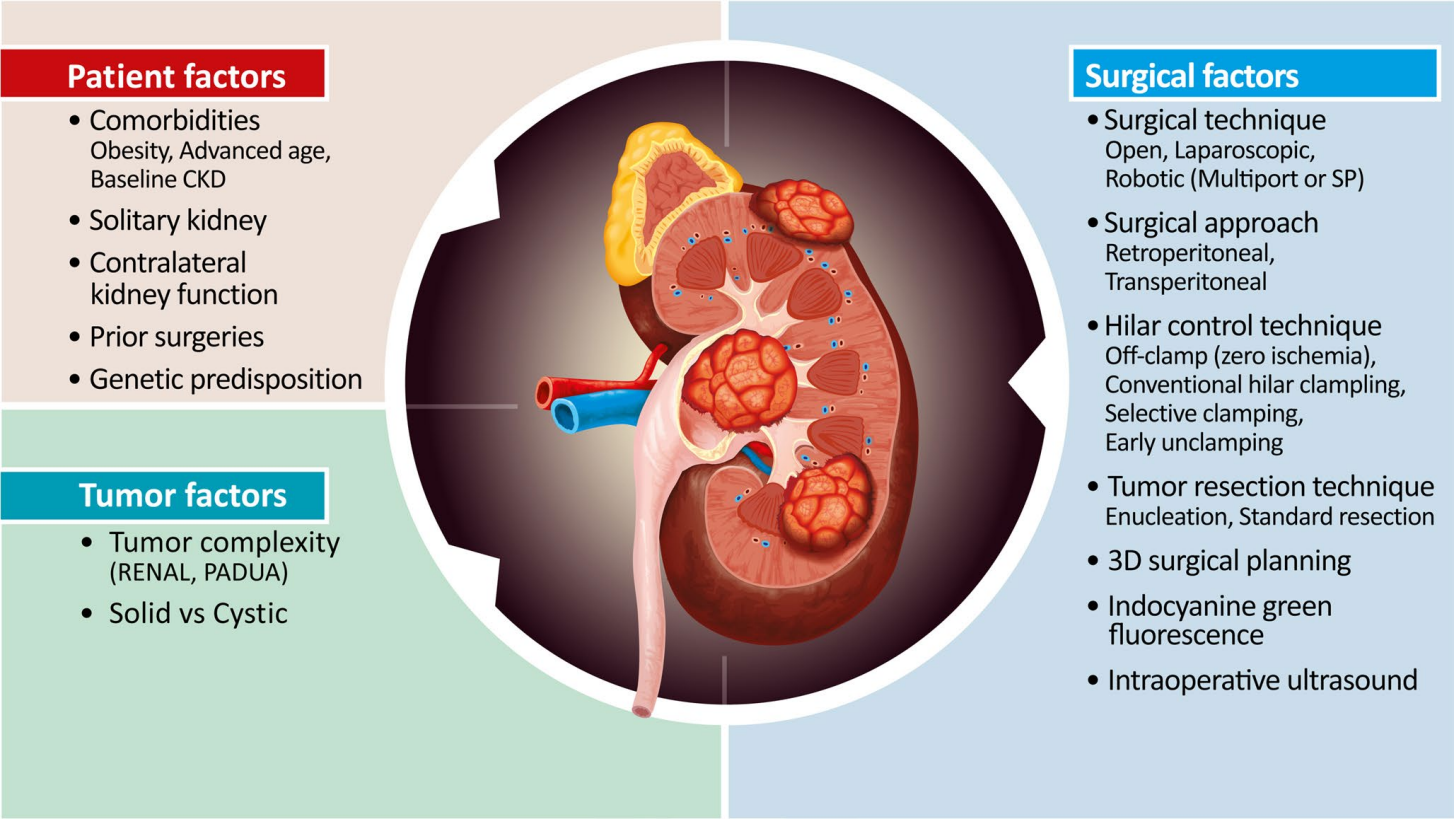
Main patient, tumor and surgical factors influencing outcomes in partial nephrectomy. Author’s original figure

Subsequent advances have expanded these early concepts into more robust 3D reconstruction and augmented-reality platforms. A 2022 systematic review and meta-analysis of 17 comparative studies involving nearly 2000 patients demonstrated that patient-specific 3D models reduce estimated blood loss, increase the use of selective ischemia and tumor enucleation, and decrease the likelihood of entering the collecting system, all without compromising margin status, complication rates, or short-term renal functional outcomes [82] (Fig. 1).

## Repair and Preservation of Renal Parenchyma

Before the adoption of modern hemostatic agents and standardized closure strategies, early experience with PN was frequently associated with significant morbidity, however, the renorraphy technique has been also a subject of debate among experts [1].

In this context, a 2019 systematic review by Bertolo et al. comparing interrupted and running renorrhaphy (124 interrupted vs. 269 continuous) found no significant differences in postoperative eGFR decline between techniques (− 4.88 mL/min vs. −3.42 mL/min, *p* = 0.14 and *p* = 0.31, respectively), indicating that the choice of suturing method can largely be guided by surgeon preference rather than differential functional impact. Additionally, in a different review by the same group, single-layer versus double layer was analyzed, demonstrating that single layer renorraphy resulted in a smaller reduction in eGFR compared with double-layer closure (− 3.19 mL/min vs. −6.07 mL/min). These findings suggest that limiting cortical suturing may offer a minimal advantage in functional preservation without increasing complications (Clavien Dindo ≥ III; *P* > 0.05), transfusion rates (pooled OR 0.67, 95% CI 0.26–1.72), overall complications (95% CI: 0.19–2.50) or positive margin rates 95% (CI: 0.01–8.22) [61, 83].

In a single-institution series of RAPN, Kaouk et al. [84] compared an interrupted bolstered capsular renorrhaphy (*n* = 65) with a continuous horizontal mattress technique (*n* = 187). The continuous approach was associated with lower estimated blood loss (approximately 248 vs. 332 mL) and a significantly reduced transfusion rate (9.0% vs. 24.6%), shorter operative time (approximately 181 vs. 219 min) and a shorter length of hospital stay (3.6 vs. 4.2 days). The overall complication rate was lower (14.4% vs. 33.8%), and fewer conversions were observed (0.5% vs. 6.1%) in the same technique. No significant differences were identified in WIT or eGFR changes.

Similarly, the impact of renorrhaphy technique on vascular complications, particularly renal artery pseudoaneurysm, was evaluated by Geldmaker et al. [83], who analyzed 563 RAPN to determine the association between inner-layer suture configuration influenced the incidence of this complication. In this study, patients received V-Loc 2 − 0 (*n* = 110), Vicryl 2 − 0 (*n* = 255), or no base suture (*n* = 198). The no-suture group had smaller tumors and therefore the shortest warm ischemia time (median 14 vs. 19–22 min, *P* < 0.001). Despite these differences, renal artery pseudoaneurysm rates were almost identical across all groups: 3.6% with V-Loc, 3.9% with Vicryl, and 3.5% with no base suture, and in a two-group analysis remained similar for base suture vs. no suture (3.8% vs. 3.5%).

Topical hemostatic agents have become an integral component of contemporary surgical management of perioperative bleeding [85, 86]. In this context, topical hemostatic agents have become an established adjunct in the surgical management of perioperative bleeding during PN. The first reported use in this setting was described by Pruthi et al. in 2004, who applied a fibrin sealant (Tisseel™) during laparoscopic PN [30]. In their 15-patient series, effective hemostasis was achieved without additional measures, with no reported bleeding events, transfusions, or urinary leaks, alongside a mean estimated blood loss of 173 mL, an operative time of 129 min and a mean length of stay of 2.7 days. More recently, a systematic review and meta-analysis, including 1,066 patients undergoing minimally invasive PN, demonstrated that sutureless techniques using hemostatic agents were associated with improved perioperative parameters, including reductions in WIT (mean difference − 6.3 min), operative time (− 19.8 min), and estimated blood loss (− 108.6 mL). Importantly, these differences were observed without a statistically significant increase in transfusion rates or hemorrhagic complications, while length of stay and short-term renal functional outcomes were comparable to those reported with standard suturing techniques [87].

## Conclusion

Minimally invasive partial nephrectomy has matured from a technically demanding alternative into the dominant nephron-sparing gold standard approach for localized renal masses. Outcomes are increasingly determined by patient and tumor factors as well as how the operation is executed.

Tumor complexity remains a key determinant of surgical strategy, while growing evidence suggests that parenchymal preservation may be more important than ischemia time alone for functional outcomes. Surgeon experience remains critical, and emerging technologies may further enhance surgical precision and decision making.

## Data Availability

No datasets were generated or analysed during the current study.
